# Supplementary material for: Identification and Genomic Characterization of Parvovirus B19V Genotype 3 Viruses from Cases of Meningoencephalitis in West Bengal, India
Source: Microbiol Spectr. 2022 Apr 12;10(2):e02251-21. doi: 10.1128/spectrum.02251-21 (PMC9045363; doi:10.1128/spectrum.02251-21)
Supplement: SUPPLEMENTAL FILE 1 — Supplemental material. Download spectrum.02251-21-s0001.pdf, PDF file, 0.1 MB [file spectrum.02251-21-s0001.pdf]

Supplementary Material

Supplementary Material for **Identification and genomic characterization of Parvovirus B19V genotype 3 viruses from cases of meningoencephalitis in West Bengal, India** by Pattabiraman et al.

Appendix 1: Metadata for complete genomes of Primate Erythroparvovirus 1 sequences (B19V) used in the study

Appendix 2: Details of cases positive for B19V by PCR

Appendix 3: Description of PCR results and recovered genomes

Appendix 4 : List of unique SNPs in B19V genomes from the study

Appendix 1 : Metadata for complete genomes of Primate Erythroparvovirus 1 sequences (B19V) used in the study

Sr. No	Accession	Release Date	Isolation Source	Collection Date	Submitters
1	KF724386.1	2014-08-11	blood	2006	Eis-Hubinger,A.M., Hofmann,J.
2	KC013303.1	2013-08-17	bone	2007	da Costa,A.C., Bendit,I., de Oliveira,A.C., Kallas,E.G., Sabino,E.C., Sanabani,S.S., de Oliveira,A.C.S.
3	KC013340.1	2013-08-17	bone	2007	da Costa,A.C., Bendit,I., de Oliveira,A.C., Kallas,E.G., Sabino,E.C., Sanabani,S.S., de Oliveira,A.C.S.
4	KC013343.1	2013-08-17	bone	2007	da Costa,A.C., Bendit,I., de Oliveira,A.C., Kallas,E.G., Sabino,E.C., Sanabani,S.S., de Oliveira,A.C.S.
5	KF724387.1	2014-08-11	blood	2008	Eis-Hubinger,A.M., Hofmann,J.
6	KC013312.1	2013-08-17	bone	2008	da Costa,A.C., Bendit,I., de Oliveira,A.C., Kallas,E.G., Sabino,E.C., Sanabani,S.S., de Oliveira,A.C.S.
7	KC013332.1	2013-08-17	bone	2008	da Costa,A.C., Bendit,I., de Oliveira,A.C., Kallas,E.G., Sabino,E.C., Sanabani,S.S., de Oliveira,A.C.S.
8	FJ591158.1	2009-01-13		2008	Chen,Z., Guan,W., Kleiboeker,S., Qiu,J.
9	MH201455.1	2019-01-21		2009	Matz,B., Kupfer,B., Kallies,R., Kulshammer,M., Flotenmeyer,M., Kreil,T.R., Eis-Hubinger,A.M.
10	MH201456.1	2019-01-21		2009	Matz,B., Kupfer,B., Kallies,R., Kulshammer,M., Flotenmeyer,M., Kreil,T.R., Eis-Hubinger,A.M.
11	KR005643.1	2015-09-30	blood	2009	Joksic,I.D., Stamenkovic,G.G., Nikolic,V.S., Siljic,M.M., Nesic,M.S., Stanojevic,M.P.
12	KC013306.1	2013-08-17	bone	2009	da Costa,A.C., Bendit,I., de Oliveira,A.C., Kallas,E.G., Sabino,E.C., Sanabani,S.S., de Oliveira,A.C.S.
13	KC013308.1	2013-08-17	bone	2009	da Costa,A.C., Bendit,I., de Oliveira,A.C., Kallas,E.G., Sabino,E.C., Sanabani,S.S., de Oliveira,A.C.S.
14	KC013310.1	2013-08-17	bone	2009	da Costa,A.C., Bendit,I., de Oliveira,A.C., Kallas,E.G., Sabino,E.C., Sanabani,S.S., de Oliveira,A.C.S.
15	KC013313.1	2013-08-17	bone	2009	da Costa,A.C., Bendit,I., de Oliveira,A.C., Kallas,E.G., Sabino,E.C., Sanabani,S.S., de Oliveira,A.C.S.
16	KC013314.1	2013-08-17	bone	2009	da Costa,A.C., Bendit,I., de Oliveira,A.C., Kallas,E.G., Sabino,E.C., Sanabani,S.S., de Oliveira,A.C.S.
17	KC013316.1	2013-08-17	bone	2009	da Costa,A.C., Bendit,I., de Oliveira,A.C., Kallas,E.G., Sabino,E.C., Sanabani,S.S., de Oliveira,A.C.S.
18	KC013321.1	2013-08-17	bone	2009	da Costa,A.C., Bendit,I., de Oliveira,A.C., Kallas,E.G., Sabino,E.C., Sanabani,S.S., de Oliveira,A.C.S.
19	KC013322.1	2013-08-17	bone	2009	da Costa,A.C., Bendit,I., de Oliveira,A.C., Kallas,E.G., Sabino,E.C., Sanabani,S.S., de

Sr. No	Accession	Release Date	Isolation Source	Collection Date	Submitters
					Oliveira,A.C.S.
20	KC013324.1	2013-08-17	bone	2009	da Costa,A.C., Bendit,I., de Oliveira,A.C., Kallas,E.G., Sabino,E.C., Sanabani,S.S., de Oliveira,A.C.S.
21	KC013328.1	2013-08-17	bone	2009	da Costa,A.C., Bendit,I., de Oliveira,A.C., Kallas,E.G., Sabino,E.C., Sanabani,S.S., de Oliveira,A.C.S.
22	KC013329.1	2013-08-17	bone	2009	da Costa,A.C., Bendit,I., de Oliveira,A.C., Kallas,E.G., Sabino,E.C., Sanabani,S.S., de Oliveira,A.C.S.
23	KC013331.1	2013-08-17	bone	2009	da Costa,A.C., Bendit,I., de Oliveira,A.C., Kallas,E.G., Sabino,E.C., Sanabani,S.S., de Oliveira,A.C.S.
24	KC013338.1	2013-08-17	bone	2009	da Costa,A.C., Bendit,I., de Oliveira,A.C., Kallas,E.G., Sabino,E.C., Sanabani,S.S., de Oliveira,A.C.S.
25	KC013344.1	2013-08-17	bone	2009	da Costa,A.C., Bendit,I., de Oliveira,A.C., Kallas,E.G., Sabino,E.C., Sanabani,S.S., de Oliveira,A.C.S.
26	KC013305.1	2013-08-17	bone	2010	da Costa,A.C., Bendit,I., de Oliveira,A.C., Kallas,E.G., Sabino,E.C., Sanabani,S.S., de Oliveira,A.C.S.
27	KC013315.1	2013-08-17	bone	2010	da Costa,A.C., Bendit,I., de Oliveira,A.C., Kallas,E.G., Sabino,E.C., Sanabani,S.S., de Oliveira,A.C.S.
28	KC013323.1	2013-08-17	bone	2010	da Costa,A.C., Bendit,I., de Oliveira,A.C., Kallas,E.G., Sabino,E.C., Sanabani,S.S., de Oliveira,A.C.S.
29	KC013325.1	2013-08-17	bone	2010	da Costa,A.C., Bendit,I., de Oliveira,A.C., Kallas,E.G., Sabino,E.C., Sanabani,S.S., de Oliveira,A.C.S.
30	KC013326.1	2013-08-17	bone	2010	da Costa,A.C., Bendit,I., de Oliveira,A.C., Kallas,E.G., Sabino,E.C., Sanabani,S.S., de Oliveira,A.C.S.
31	KC013327.1	2013-08-17	bone	2010	da Costa,A.C., Bendit,I., de Oliveira,A.C., Kallas,E.G., Sabino,E.C., Sanabani,S.S., de Oliveira,A.C.S.
32	KC013333.1	2013-08-17	bone	2010	da Costa,A.C., Bendit,I., de Oliveira,A.C., Kallas,E.G., Sabino,E.C., Sanabani,S.S., de Oliveira,A.C.S.
33	KC013346.1	2013-08-17	bone	2010	da Costa,A.C., Bendit,I., de Oliveira,A.C., Kallas,E.G., Sabino,E.C., Sanabani,S.S., de Oliveira,A.C.S.
34	KC013351.1	2013-08-17	bone	2010	da Costa,A.C., Bendit,I., de Oliveira,A.C., Kallas,E.G., Sabino,E.C., Sanabani,S.S., de Oliveira,A.C.S.
35	KR005640.1	2015-09-30	blood	2011	Stamenkovic,G.G., Nikolic,V.S., Siljic,M.M., Nesic,M.S., Stanojevic,M.P.
36	KR005641.1	2015-09-30	blood	2011	Stamenkovic,G.G., Nikolic,V.S., Siljic,M.M., Nesic,M.S., Stanojevic,M.P.
37	KR005642.1	2015-09-30	blood	2011	Stamenkovic,G.G., Nikolic,V.S., Siljic,M.M., Nesic,M.S., Stanojevic,M.P.
38	KR005644.1	2015-09-30	blood	2012	Knezevic,A.M., Stamenkovic,G.G.,

Sr. No	Accession	Release Date	Isolation Source	Collection Date	Submitters
					Nikolic,V.S., Siljic,M.M., Nesic,M.S., Stanojevic,M.P.
39	KM393163.1	2014-10-12	blood	2013	Qiu,Y., Kleiboeker,S., Li,B.
40	KM393164.1	2014-10-12	blood	2013	Qiu,Y., Kleiboeker,S., Li,B.
41	KM393165.1	2014-10-12	blood	2013	Qiu,Y., Kleiboeker,S., Li,B.
42	KM393166.1	2014-10-12	blood	2013	Qiu,Y., Kleiboeker,S., Li,B.
43	KM393167.1	2014-10-12	blood	2013	Qiu,Y., Kleiboeker,S., Li,B.
44	KM393168.1	2014-10-12	blood	2013	Qiu,Y., Kleiboeker,S., Li,B.
45	KM393169.1	2014-10-12	blood	2013	Qiu,Y., Kleiboeker,S., Li,B.
46	KT310174.1	2015-12-06		2014	Zhang,L., Cai,C., Pan,F., Hong,L., Luo,X., Hu,S., Xu,J., Chen,Z.
47	MT988397.1	2021-04-27	bone	2015	Toppinen,M., Sajantila,A., Pratas,D., Hedman,K., Perdomo,M.F.
48	MT988398.1	2021-04-27	bone	2015	Toppinen,M., Sajantila,A., Pratas,D., Hedman,K., Perdomo,M.F.
49	MT988399.1	2021-04-27	bone	2015	Toppinen,M., Sajantila,A., Pratas,D., Hedman,K., Perdomo,M.F.
50	MT988400.1	2021-04-27	bone	2015	Toppinen,M., Sajantila,A., Pratas,D., Hedman,K., Perdomo,M.F.
51	MT988401.1	2021-04-27	bone	2015	Toppinen,M., Sajantila,A., Pratas,D., Hedman,K., Perdomo,M.F.
52	MT988402.1	2021-04-27	bone	2015	Toppinen,M., Sajantila,A., Pratas,D., Hedman,K., Perdomo,M.F.
53	MT988403.1	2021-04-27	bone	2015	Toppinen,M., Sajantila,A., Pratas,D., Hedman,K., Perdomo,M.F.
54	MT682520.1	2020-08-18		2015	Pratas,D., Toppinen,M., Pyoria,L., Hedman,K., Sajantilla,A., Perdomo,M.F.
55	MT410184.1	2020-08-04		2015	Toppinen,M., Pratas,D., Vaisanen,E., Soderlund-Venermo,M., Hedman,K., Perdomo,M.F., Sajantila,A.
56	MT410185.1	2020-08-04		2015	Toppinen,M., Pratas,D., Vaisanen,E., Soderlund-Venermo,M., Hedman,K., Perdomo,M.F., Sajantila,A.
57	MT410186.1	2020-08-04		2015	Toppinen,M., Pratas,D., Vaisanen,E., Soderlund-Venermo,M., Hedman,K., Perdomo,M.F., Sajantila,A.
58	MT410187.1	2020-08-04		2015	Toppinen,M., Pratas,D., Vaisanen,E., Soderlund-Venermo,M., Hedman,K., Perdomo,M.F., Sajantila,A.
59	MT410188.1	2020-08-04		2015	Toppinen,M., Pratas,D., Vaisanen,E., Soderlund-Venermo,M., Hedman,K., Perdomo,M.F., Sajantila,A.
60	MT410189.1	2020-08-04		2015	Toppinen,M., Pratas,D., Vaisanen,E., Soderlund-Venermo,M., Hedman,K., Perdomo,M.F., Sajantila,A.
61	MT410190.1	2020-08-04		2015	Toppinen,M., Pratas,D., Vaisanen,E., Soderlund-Venermo,M., Hedman,K., Perdomo,M.F., Sajantila,A.
62	FN598217.1	2009-12-01		2002-02-22	Op de Beeck,A., Caillet-Fauquet,P., Baurin,S., Laub,R.
63	JN211125.1	2012-08-14		2003-03	Molenaar-de Backer,M.W., Lukashov,V.V., van

Sr. No	Accession	Release Date	Isolation Source	Collection Date	Submitters
					Binnendijk,R.S., Boot,H.J., Zaaijer,H.L., Molenaar-de Backer,M.W.A., van Swieten,P.
64	JN211127.1	2012-08-14		2003-04	Molenaar-de Backer,M.W., Lukashov,V.V., van Binnendijk,R.S., Boot,H.J., Zaaijer,H.L., Molenaar-de Backer,M.W.A., van Swieten,P.
65	JN211154.1	2012-08-14		2003-04	Molenaar-de Backer,M.W., Lukashov,V.V., van Binnendijk,R.S., Boot,H.J., Zaaijer,H.L., Molenaar-de Backer,M.W.A., van Swieten,P.
66	JN211155.1	2012-08-14		2003-04	Molenaar-de Backer,M.W., Lukashov,V.V., van Binnendijk,R.S., Boot,H.J., Zaaijer,H.L., Molenaar-de Backer,M.W.A., van Swieten,P.
67	JN211157.1	2012-08-14		2003-04	Molenaar-de Backer,M.W., Lukashov,V.V., van Binnendijk,R.S., Boot,H.J., Zaaijer,H.L., Molenaar-de Backer,M.W.A., van Swieten,P.
68	JN211185.1	2012-08-14		2003-04	Molenaar-de Backer,M.W., Lukashov,V.V., van Binnendijk,R.S., Boot,H.J., Zaaijer,H.L., Molenaar-de Backer,M.W.A., van Swieten,P.
69	JN211167.1	2012-08-14		2003-05	Molenaar-de Backer,M.W., Lukashov,V.V., van Binnendijk,R.S., Boot,H.J., Zaaijer,H.L., Molenaar-de Backer,M.W.A., van Swieten,P.
70	JN211170.1	2012-08-14		2003-05	Molenaar-de Backer,M.W., Lukashov,V.V., van Binnendijk,R.S., Boot,H.J., Zaaijer,H.L., Molenaar-de Backer,M.W.A., van Swieten,P.
71	JN211130.1	2012-08-14		2003-07	Molenaar-de Backer,M.W., Lukashov,V.V., van Binnendijk,R.S., Boot,H.J., Zaaijer,H.L., Molenaar-de Backer,M.W.A., van Swieten,P.
72	JN211163.1	2012-08-14		2003-07	Molenaar-de Backer,M.W., Lukashov,V.V., van Binnendijk,R.S., Boot,H.J., Zaaijer,H.L., Molenaar-de Backer,M.W.A., van Swieten,P.
73	JN211180.1	2012-08-14		2003-08	Molenaar-de Backer,M.W., Lukashov,V.V., van Binnendijk,R.S., Boot,H.J., Zaaijer,H.L., Molenaar-de Backer,M.W.A., van Swieten,P.
74	JN211138.1	2012-08-14		2003-12	Molenaar-de Backer,M.W., Lukashov,V.V., van Binnendijk,R.S., Boot,H.J., Zaaijer,H.L., Molenaar-de Backer,M.W.A., van Swieten,P.
75	JN211139.1	2012-08-14		2003-12	Molenaar-de Backer,M.W., Lukashov,V.V., van Binnendijk,R.S., Boot,H.J., Zaaijer,H.L., Molenaar-de Backer,M.W.A., van Swieten,P.
76	JN211183.1	2012-08-14		2003-12	Molenaar-de Backer,M.W., Lukashov,V.V., van Binnendijk,R.S., Boot,H.J., Zaaijer,H.L., Molenaar-de Backer,M.W.A., van Swieten,P.
77	JN211129.1	2012-08-14		2004-01	Molenaar-de Backer,M.W., Lukashov,V.V., van Binnendijk,R.S., Boot,H.J., Zaaijer,H.L., Molenaar-de Backer,M.W.A., van Swieten,P.
78	JN211181.1	2012-08-14		2004-01	Molenaar-de Backer,M.W., Lukashov,V.V., van Binnendijk,R.S., Boot,H.J., Zaaijer,H.L., Molenaar-de Backer,M.W.A., van Swieten,P.
79	JN211144.1	2012-08-14		2004-02	Molenaar-de Backer,M.W., Lukashov,V.V., van Binnendijk,R.S., Boot,H.J., Zaaijer,H.L., Molenaar-de Backer,M.W.A., van Swieten,P.
80	JN211150.1	2012-08-14		2004-03	Molenaar-de Backer,M.W., Lukashov,V.V., van Binnendijk,R.S., Boot,H.J., Zaaijer,H.L., Molenaar-de Backer,M.W.A., van Swieten,P.

Sr. No	Accession	Release Date	Isolation Source	Collection Date	Submitters
81	JN211123.1	2012-08-14		2004-04	Molenaar-de Backer,M.W., Lukashov,V.V., van Binnendijk,R.S., Boot,H.J., Zaaijer,H.L., Molenaar-de Backer,M.W.A., van Swieten,P.
82	JN211151.1	2012-08-14		2004-04	Molenaar-de Backer,M.W., Lukashov,V.V., van Binnendijk,R.S., Boot,H.J., Zaaijer,H.L., Molenaar-de Backer,M.W.A., van Swieten,P.
83	JN211184.1	2012-08-14		2004-04	Molenaar-de Backer,M.W., Lukashov,V.V., van Binnendijk,R.S., Boot,H.J., Zaaijer,H.L., Molenaar-de Backer,M.W.A., van Swieten,P.
84	JN211153.1	2012-08-14		2004-06	Molenaar-de Backer,M.W., Lukashov,V.V., van Binnendijk,R.S., Boot,H.J., Zaaijer,H.L., Molenaar-de Backer,M.W.A., van Swieten,P.
85	JN211182.1	2012-08-14		2004-06	Molenaar-de Backer,M.W., Lukashov,V.V., van Binnendijk,R.S., Boot,H.J., Zaaijer,H.L., Molenaar-de Backer,M.W.A., van Swieten,P.
86	JN211134.1	2012-08-14		2004-07	Molenaar-de Backer,M.W., Lukashov,V.V., van Binnendijk,R.S., Boot,H.J., Zaaijer,H.L., Molenaar-de Backer,M.W.A., van Swieten,P.
87	JN211176.1	2012-08-14		2004-07	Molenaar-de Backer,M.W., Lukashov,V.V., van Binnendijk,R.S., Boot,H.J., Zaaijer,H.L., Molenaar-de Backer,M.W.A., van Swieten,P.
88	JN211160.1	2012-08-14		2004-09	Molenaar-de Backer,M.W., Lukashov,V.V., van Binnendijk,R.S., Boot,H.J., Zaaijer,H.L., Molenaar-de Backer,M.W.A., van Swieten,P.
89	JN211162.1	2012-08-14		2004-10	Molenaar-de Backer,M.W., Lukashov,V.V., van Binnendijk,R.S., Boot,H.J., Zaaijer,H.L., Molenaar-de Backer,M.W.A., van Swieten,P.
90	JN211149.1	2012-08-14		2004-11	Molenaar-de Backer,M.W., Lukashov,V.V., van Binnendijk,R.S., Boot,H.J., Zaaijer,H.L., Molenaar-de Backer,M.W.A., van Swieten,P.
91	JN211177.1	2012-08-14		2004-12	Molenaar-de Backer,M.W., Lukashov,V.V., van Binnendijk,R.S., Boot,H.J., Zaaijer,H.L., Molenaar-de Backer,M.W.A., van Swieten,P.
92	JN211121.1	2012-08-14		2006-01	Molenaar-de Backer,M.W., Lukashov,V.V., van Binnendijk,R.S., Boot,H.J., Zaaijer,H.L., Molenaar-de Backer,M.W.A., van Swieten,P.
93	JN211131.1	2012-08-14		2006-02	Molenaar-de Backer,M.W., Lukashov,V.V., van Binnendijk,R.S., Boot,H.J., Zaaijer,H.L., Molenaar-de Backer,M.W.A., van Swieten,P.
94	JN211132.1	2012-08-14		2006-02	Molenaar-de Backer,M.W., Lukashov,V.V., van Binnendijk,R.S., Boot,H.J., Zaaijer,H.L., Molenaar-de Backer,M.W.A., van Swieten,P.
95	JN211128.1	2012-08-14		2006-03	Molenaar-de Backer,M.W., Lukashov,V.V., van Binnendijk,R.S., Boot,H.J., Zaaijer,H.L., Molenaar-de Backer,M.W.A., van Swieten,P.
96	JN211145.1	2012-08-14		2006-03	Molenaar-de Backer,M.W., Lukashov,V.V., van Binnendijk,R.S., Boot,H.J., Zaaijer,H.L., Molenaar-de Backer,M.W.A., van Swieten,P.
97	JN211169.1	2012-08-14		2006-03	Molenaar-de Backer,M.W., Lukashov,V.V., van Binnendijk,R.S., Boot,H.J., Zaaijer,H.L., Molenaar-de Backer,M.W.A., van Swieten,P.
98	JN211143.1	2012-08-14		2006-04	Molenaar-de Backer,M.W., Lukashov,V.V., van Binnendijk,R.S., Boot,H.J., Zaaijer,H.L.,

Sr. No	Accession	Release Date	Isolation Source	Collection Date	Submitters
					Molenaar-de Backer,M.W.A., van Swieten,P.
99	JN211166.1	2012-08-14		2006-04	Molenaar-de Backer,M.W., Lukashov,V.V., van Binnendijk,R.S., Boot,H.J., Zaaijer,H.L., Molenaar-de Backer,M.W.A., van Swieten,P.
100	JN211164.1	2012-08-14		2006-05	Molenaar-de Backer,M.W., Lukashov,V.V., van Binnendijk,R.S., Boot,H.J., Zaaijer,H.L., Molenaar-de Backer,M.W.A., van Swieten,P.
101	JN211141.1	2012-08-14		2006-06	Molenaar-de Backer,M.W., Lukashov,V.V., van Binnendijk,R.S., Boot,H.J., Zaaijer,H.L., Molenaar-de Backer,M.W.A., van Swieten,P.
102	JN211147.1	2012-08-14		2006-06	Molenaar-de Backer,M.W., Lukashov,V.V., van Binnendijk,R.S., Boot,H.J., Zaaijer,H.L., Molenaar-de Backer,M.W.A., van Swieten,P.
103	JN211161.1	2012-08-14		2006-07	Molenaar-de Backer,M.W., Lukashov,V.V., van Binnendijk,R.S., Boot,H.J., Zaaijer,H.L., Molenaar-de Backer,M.W.A., van Swieten,P.
104	JN211179.1	2012-08-14		2006-07	Molenaar-de Backer,M.W., Lukashov,V.V., van Binnendijk,R.S., Boot,H.J., Zaaijer,H.L., Molenaar-de Backer,M.W.A., van Swieten,P.
105	JN211173.1	2012-08-14		2006-09	Molenaar-de Backer,M.W., Lukashov,V.V., van Binnendijk,R.S., Boot,H.J., Zaaijer,H.L., Molenaar-de Backer,M.W.A., van Swieten,P.
106	FJ265736.1	2008-10-28		2007-02	Rinckel,L.A., Buno,B.R., Gierman,T.M., Lee,D.C.
107	JN211148.1	2012-08-14		2008-05	Molenaar-de Backer,M.W., Lukashov,V.V., van Binnendijk,R.S., Boot,H.J., Zaaijer,H.L., Molenaar-de Backer,M.W.A., van Swieten,P.
108	JN211136.1	2012-08-14		2008-06	Molenaar-de Backer,M.W., Lukashov,V.V., van Binnendijk,R.S., Boot,H.J., Zaaijer,H.L., Molenaar-de Backer,M.W.A., van Swieten,P.
109	JN211168.1	2012-08-14		2008-06	Molenaar-de Backer,M.W., Lukashov,V.V., van Binnendijk,R.S., Boot,H.J., Zaaijer,H.L., Molenaar-de Backer,M.W.A., van Swieten,P.
110	JN211135.1	2012-08-14		2008-07	Molenaar-de Backer,M.W., Lukashov,V.V., van Binnendijk,R.S., Boot,H.J., Zaaijer,H.L., Molenaar-de Backer,M.W.A., van Swieten,P.
111	JN211175.1	2012-08-14		2008-07	Molenaar-de Backer,M.W., Lukashov,V.V., van Binnendijk,R.S., Boot,H.J., Zaaijer,H.L., Molenaar-de Backer,M.W.A., van Swieten,P.
112	JN211178.1	2012-08-14		2008-10	Molenaar-de Backer,M.W., Lukashov,V.V., van Binnendijk,R.S., Boot,H.J., Zaaijer,H.L., Molenaar-de Backer,M.W.A., van Swieten,P.
113	JN211171.1	2012-08-14		2008-12	Molenaar-de Backer,M.W., Lukashov,V.V., van Binnendijk,R.S., Boot,H.J., Zaaijer,H.L., Molenaar-de Backer,M.W.A., van Swieten,P.
114	JN211172.1	2012-08-14		2008-12	Molenaar-de Backer,M.W., Lukashov,V.V., van Binnendijk,R.S., Boot,H.J., Zaaijer,H.L., Molenaar-de Backer,M.W.A., van Swieten,P.
115	JN211124.1	2012-08-14		2009-01	Molenaar-de Backer,M.W., Lukashov,V.V., van Binnendijk,R.S., Boot,H.J., Zaaijer,H.L., Molenaar-de Backer,M.W.A., van Swieten,P.
116	JN211122.1	2012-08-14		2009-02	Molenaar-de Backer,M.W., Lukashov,V.V., van Binnendijk,R.S., Boot,H.J., Zaaijer,H.L.,

Sr. No	Accession	Release Date	Isolation Source	Collection Date	Submitters
					Molenaar-de Backer,M.W.A., van Swieten,P.
117	JN211126.1	2012-08-14		2009-02	Molenaar-de Backer,M.W., Lukashov,V.V., van Binnendijk,R.S., Boot,H.J., Zaaijer,H.L., Molenaar-de Backer,M.W.A., van Swieten,P.
118	JN211133.1	2012-08-14		2009-03	Molenaar-de Backer,M.W., Lukashov,V.V., van Binnendijk,R.S., Boot,H.J., Zaaijer,H.L., Molenaar-de Backer,M.W.A., van Swieten,P.
119	JN211140.1	2012-08-14		2009-04	Molenaar-de Backer,M.W., Lukashov,V.V., van Binnendijk,R.S., Boot,H.J., Zaaijer,H.L., Molenaar-de Backer,M.W.A., van Swieten,P.
120	JN211142.1	2012-08-14		2009-04	Molenaar-de Backer,M.W., Lukashov,V.V., van Binnendijk,R.S., Boot,H.J., Zaaijer,H.L., Molenaar-de Backer,M.W.A., van Swieten,P.
121	JN211137.1	2012-08-14		2009-05	Molenaar-de Backer,M.W., Lukashov,V.V., van Binnendijk,R.S., Boot,H.J., Zaaijer,H.L., Molenaar-de Backer,M.W.A., van Swieten,P.
122	JN211146.1	2012-08-14		2009-05	Molenaar-de Backer,M.W., Lukashov,V.V., van Binnendijk,R.S., Boot,H.J., Zaaijer,H.L., Molenaar-de Backer,M.W.A., van Swieten,P.
123	JN211152.1	2012-08-14		2009-05	Molenaar-de Backer,M.W., Lukashov,V.V., van Binnendijk,R.S., Boot,H.J., Zaaijer,H.L., Molenaar-de Backer,M.W.A., van Swieten,P.
124	JN211156.1	2012-08-14		2009-07	Molenaar-de Backer,M.W., Lukashov,V.V., van Binnendijk,R.S., Boot,H.J., Zaaijer,H.L., Molenaar-de Backer,M.W.A., van Swieten,P.
125	JN211159.1	2012-08-14		2009-07	Molenaar-de Backer,M.W., Lukashov,V.V., van Binnendijk,R.S., Boot,H.J., Zaaijer,H.L., Molenaar-de Backer,M.W.A., van Swieten,P.
126	HQ340601.1	2010-11-24	heart	2009-07	Utta,F., Kandolf,R., Bock,C.T.
127	HQ340602.1	2010-11-24	heart	2009-07	Utta,F., Kandolf,R., Bock,C.T.
128	JN211158.1	2012-08-14		2009-08	Molenaar-de Backer,M.W., Lukashov,V.V., van Binnendijk,R.S., Boot,H.J., Zaaijer,H.L., Molenaar-de Backer,M.W.A., van Swieten,P.
129	JN211165.1	2012-08-14		2009-08	Molenaar-de Backer,M.W., Lukashov,V.V., van Binnendijk,R.S., Boot,H.J., Zaaijer,H.L., Molenaar-de Backer,M.W.A., van Swieten,P.
130	JN211174.1	2012-08-14		2009-08	Molenaar-de Backer,M.W., Lukashov,V.V., van Binnendijk,R.S., Boot,H.J., Zaaijer,H.L., Molenaar-de Backer,M.W.A., van Swieten,P.
131	MH151117.1	2018-12-24		2017-07	Jia,J., Zhong,Y., Zhao,X., Guo,Y., Ma,Y., Zhang,J.
132	NC_000883.2	1999-08-02			Zhi,N., Zadori,Z., Brown,K.E., Tijssen,P.
133	MP326641.1	2020-05-15			GAO,K., LINNEN,J.M., NORTON,K.C., GORDON,P.C., DO,D., LE,T.N.
134	MP327831.1	2020-05-15			GAO,K., LINNEN,J.M., NORTON,K.C., GORDON,P.C., DO,D., LE,T.N.
135	MK989716.1	2019-08-17			Phan,M.V.T., Agoti,C.N., Nokes,D.J., Cotten,M.
136	MA897859.1	2019-07-31			Le,T.N., Do,D., Gordon,P.C., Linnen,J.M., Norton,K.C., Gao,K.
137	LP830882.1	2018-04-29			CARRICK,J.
138	LP830895.1	2018-04-29			CARRICK,J.

Sr. No	Accession	Release Date	Isolation Source	Collection Date	Submitters
139	LZ247063.1	2018-02-28			Le,T.N., Gao,K., Linnen,J.M., Norton,K.C., Gordon,P.C., Do,D.
140	KX752821.1	2017-08-19			Fryer,J.F., Fritzsche,M., Holmes,N., Nordgren,A.
141	LX096830.1	2017-07-29			Kaneko,A., Ran,G., Aoyagi,K.
142	MS987313.1	2017-07-12			CARRICK,J.
143	MS987326.1	2017-07-12			CARRICK,J.
144	LQ249377.1	2016-10-05			GAO,K., LINNEN,J., NORTON,K.C., DAT,D., LE,T.N., GORDON,P.
145	LN680968.2	2016-02-01			Ivanova,S.K., Mihneva,Z.G., Toshev,A.K., Kovaleva,V.P., Andonova,L.G., Muller,C.P., Huebschen,J.M., Huebschen,J.
146	HW766536.1	2015-02-24			Gordon,P.C., Le,T.N., Gao,K., Linnen,J.M., Do,D., Norton,K.C.
147	KM065414.1	2014-08-26	blood		Trosemeier,J.H., Branting,A., Lukashov,V.V., Blumel,J., Baylis,S.A., Bluemel,J., Troesemeier,J.-H.
148	KM065415.1	2014-08-26	blood		Trosemeier,J.H., Branting,A., Lukashov,V.V., Blumel,J., Baylis,S.A., Bluemel,J., Troesemeier,J.-H.
149	JB292192.1	2013-09-25			HEWITT,C., SAMULSKI,R.J.
150	JB151806.1	2013-04-25			CARRICK,J.M.
151	JB151819.1	2013-04-25			CARRICK,J.M.
152	JA829545.1	2013-01-29			GAO,K., LINNEN,J.M., NORTON,K.C., GORDON,P.C., DO,D., LE,T.N.
153	FW377125.1	2010-09-30			Rinkeru,L.A., Buno,B., Bimusu,B., Glenn,C., Giaman,T.M., Lee,D.C.
154	FW377252.1	2010-09-30			Rinkeru,L.A., Buno,B., Bimusu,B., Glenn,C., Giaman,T.M., Lee,D.C.
155	FW377253.1	2010-09-30			Rinkeru,L.A., Buno,B., Bimusu,B., Glenn,C., Giaman,T.M., Lee,D.C.
156	FW377254.1	2010-09-30			Rinkeru,L.A., Buno,B., Bimusu,B., Glenn,C., Giaman,T.M., Lee,D.C.
157	FW377255.1	2010-09-30			Rinkeru,L.A., Buno,B., Bimusu,B., Glenn,C., Giaman,T.M., Lee,D.C.
158	FW377256.1	2010-09-30			Rinkeru,L.A., Buno,B., Bimusu,B., Glenn,C., Giaman,T.M., Lee,D.C.
159	FW377257.1	2010-09-30			Rinkeru,L.A., Buno,B., Bimusu,B., Glenn,C., Giaman,T.M., Lee,D.C.
160	FN669502.1	2010-05-20			Dina,J., Villedieu,F., Labombarda,F., Freymuth,F., de la Gastine,G., Jokic,M., Vabret,A.
161	FN669503.1	2010-05-20			Dina,J., Villedieu,F., Labombarda,F., Freymuth,F., de la Gastine,G., Jokic,M., Vabret,A.
162	FN669504.1	2010-05-20			Dina,J., Villedieu,F., Labombarda,F., Freymuth,F., de la Gastine,G., Jokic,M., Vabret,A.
163	FN669505.1	2010-05-20	blood		Dina,J., Villedieu,F., Labombarda,F., Freymuth,F., de la Gastine,G., Jokic,M., Vabret,A.

Sr. No	Accession	Release Date	Isolation Source	Collection Date	Submitters
164	FN669506.1	2010-05-20			Dina,J., Villedieu,F., Labombarda,F., Freymuth,F., de la Gastine,G., Jokic,M., Vabret,A.
165	FN669507.1	2010-05-20			Dina,J., Villedieu,F., Labombarda,F., Freymuth,F., de la Gastine,G., Jokic,M., Vabret,A.
166	FV537070.1	2010-03-18			Millar,D.S., Meruki,J.R.
167	FV537071.1	2010-03-18			Millar,D.S., Meruki,J.R.
168	FV537072.1	2010-03-18			Millar,D.S., Meruki,J.R.
169	FV537073.1	2010-03-18			Millar,D.S., Meruki,J.R.
170	AB550331.1	2010-03-13			Tsujikawa,M., Nishigaki,H., Yoshikawa,M., Kubo,J.A., Shimamura,Y., Urayama,T., Hattori,S., Yunoki,M., Ikuta,K.
171	FN598218.1	2009-12-01			Op de Beeck,A., Caillet-Fauquet,P., Baurin,S., Laub,R.
172	GM703832.1	2008-12-19			Lee,D.C., Gierman,T.M., Glenn,C., Beames,B., Buno,B., Rinckel,L.A.
173	GM703959.1	2008-12-19			Lee,D.C., Gierman,T.M., Glenn,C., Beames,B., Buno,B., Rinckel,L.A.
174	GM703960.1	2008-12-19			Lee,D.C., Gierman,T.M., Glenn,C., Beames,B., Buno,B., Rinckel,L.A.
175	GM703961.1	2008-12-19			Lee,D.C., Gierman,T.M., Glenn,C., Beames,B., Buno,B., Rinckel,L.A.
176	GM703962.1	2008-12-19			Lee,D.C., Gierman,T.M., Glenn,C., Beames,B., Buno,B., Rinckel,L.A.
177	GM703963.1	2008-12-19			Lee,D.C., Gierman,T.M., Glenn,C., Beames,B., Buno,B., Rinckel,L.A.
178	GM703964.1	2008-12-19			Lee,D.C., Gierman,T.M., Glenn,C., Beames,B., Buno,B., Rinckel,L.A.
179	FB715682.1	2008-11-19			Wong,S., Young,N.S., Zhi,N., Brown,K.
180	FB715683.1	2008-11-19			Wong,S., Young,N.S., Zhi,N., Brown,K.
181	EF216869.1	2007-02-14	blood		Baylis,S.A.
182	DQ408301.1	2006-03-12			Schneider,B., Hone,A., Tolba,R.H., Fischer,H.P., Blumel,J., Eis-Hubinger,A.M.
183	DQ408302.1	2006-03-12			Schneider,B., Hone,A., Tolba,R.H., Fischer,H.P., Blumel,J., Eis-Hubinger,A.M.
184	DQ408303.1	2006-03-12			Schneider,B., Hone,A., Tolba,R.H., Fischer,H.P., Blumel,J., Eis-Hubinger,A.M.
185	DQ408304.1	2006-03-12			Schneider,B., Hone,A., Tolba,R.H., Fischer,H.P., Blumel,J., Eis-Hubinger,A.M.
186	DQ408305.1	2006-03-12			Schneider,B., Hone,A., Tolba,R.H., Fischer,H.P., Blumel,J., Eis-Hubinger,A.M.
187	DQ357064.1	2006-02-05			Toan,N.L., Duechting,A., Kremsner,P.G., Song,L.H., Ebinger,M., Aberle,S., Binh,V.Q., Duy,D.N., Torresi,J., Kandolf,R., Bock,C.T., Bock,T.C.
188	DQ357065.1	2006-02-05			Toan,N.L., Duechting,A., Kremsner,P.G., Song,L.H., Ebinger,M., Aberle,S., Binh,V.Q., Duy,D.N., Torresi,J., Kandolf,R., Bock,C.T., Bock,T.C.
189	DQ333426.1	2006-01-08			Schneider,B., Hone,A., Tolba,R.H.,

Sr. No	Accession	Release Date	Isolation Source	Collection Date	Submitters
					Fischer,H.P., Blumel,J., Eis-Hubinger,A.M.
190	DQ333427.1	2006-01-08			Schneider,B., Hone,A., Tolba,R.H., Fischer,H.P., Blumel,J., Eis-Hubinger,A.M.
191	DQ333428.1	2006-01-08			Schneider,B., Hone,A., Tolba,R.H., Fischer,H.P., Blumel,J., Eis-Hubinger,A.M.
192	DQ293995.2	2005-12-11	blood		Parsyan,A., Kerr,S., Owusu-Ofori,S., Elliott,G., Allain,J.P., Parsyan,A.E., Thomas,I., Laub,R., Allain,J.-P., Szmaragd,C., Candotti,D.
193	DQ234769.2	2005-11-02	blood		Parsyan,A., Kerr,S., Owusu-Ofori,S., Elliott,G., Allain,J.P., Szmaragd,C., Candotti,D., Parsyan,A.E., Addo-Yobo,E., Akpene,H., Sarkodie,F., Allain,J.-P.
194	DQ234771.2	2005-11-02	blood		Parsyan,A., Kerr,S., Owusu-Ofori,S., Elliott,G., Allain,J.P., Szmaragd,C., Candotti,D., Parsyan,A.E., Addo-Yobo,E., Akpene,H., Sarkodie,F., Allain,J.-P.
195	DQ234775.2	2005-11-02	blood		Parsyan,A., Szmaragd,C., Allain,J.P., Candotti,D., Parsyan,A.E., Addo-Yobo,E., Owusu-Ofori,S., Akpene,H., Sarkodie,F., Allain,J.-P.
196	DQ234778.2	2005-11-02	blood		Parsyan,A., Kerr,S., Owusu-Ofori,S., Elliott,G., Allain,J.P., Szmaragd,C., Candotti,D., Parsyan,A.E., Addo-Yobo,E., Akpene,H., Sarkodie,F., Allain,J.-P.
197	DQ234779.2	2005-11-02	blood		Parsyan,A., Kerr,S., Owusu-Ofori,S., Elliott,G., Allain,J.P., Szmaragd,C., Candotti,D., Parsyan,A.E., Addo-Yobo,E., Akpene,H., Sarkodie,F., Allain,J.-P.
198	DQ225148.1	2005-10-26			Toan,N.L., Duechting,A., Kremsner,P.G., Song,L.H., Ebinger,M., Aberle,S., Binh,V.Q., Duy,D.N., Torresi,J., Kandolf,R., Bock,C.T., Kaiser,H., Klingel,K., Bock,T.
199	DQ225149.1	2005-10-26			Toan,N.L., Duechting,A., Kremsner,P.G., Song,L.H., Ebinger,M., Aberle,S., Binh,V.Q., Duy,D.N., Torresi,J., Kandolf,R., Bock,C.T., Kaiser,H., Klingel,K., Bock,T.
200	DQ225150.1	2005-10-26			Toan,N.L., Duechting,A., Kremsner,P.G., Song,L.H., Ebinger,M., Aberle,S., Binh,V.Q., Duy,D.N., Torresi,J., Kandolf,R., Bock,C.T., Kaiser,H., Klingel,K., Bock,T.
201	DQ225151.1	2005-10-26			Toan,N.L., Duechting,A., Kremsner,P.G., Song,L.H., Ebinger,M., Aberle,S., Binh,V.Q., Duy,D.N., Torresi,J., Kandolf,R., Bock,C.T., Kaiser,H., Klingel,K., Bock,T.
202	AY903437.2	2005-10-01			Blumel,J., Eis-Hubinger,A.M., Stuhler,A., Bonsch,C., Gessner,M., Lower,J.
203	AY647977.1	2004-07-07	blood		Keller,L.W., Barbosa,M.L., Durigon,E.L.
204	AJ781031.1	2004-07-01			Plentz,A., Hahn,J., Holler,E., Jilg,W., Modrow,S.
205	AJ781032.1	2004-07-01			Plentz,A., Hahn,J., Holler,E., Jilg,W., Modrow,S.
206	AJ781033.1	2004-07-01			Plentz,A., Hahn,J., Holler,E., Jilg,W.,

Sr. No	Accession	Release Date	Isolation Source	Collection Date	Submitters
					Modrow,S.
207	AJ781034.1	2004-07-01			Plentz,A., Hahn,J., Holler,E., Jilg,W., Modrow,S.
208	AJ781035.1	2004-07-01			Plentz,A., Hahn,J., Holler,E., Jilg,W., Modrow,S.
209	AJ781036.1	2004-07-01			Plentz,A., Hahn,J., Holler,E., Jilg,W., Modrow,S.
210	AJ781037.1	2004-07-01			Plentz,A., Hahn,J., Holler,E., Jilg,W., Modrow,S.
211	AJ781038.1	2004-07-01			Plentz,A., Hahn,J., Holler,E., Jilg,W., Modrow,S.
212	AJ717293.1	2004-05-26	blood		Liefeldt,L., Plentz,A., Raab,U., Klempa,B., Kershaw,O., Endres,A.S., Neumayer,H.H., Meisel,H., Modrow,S.
213	AY582124.2	2004-04-11			Parsyan,A., Kerr,S., Owusu-Ofori,S., Elliott,G., Allain,J.P., Szmaragd,C., Allain,J.-P., Candotti,D., Parsyan,A.E.
214	AY582125.2	2004-04-11			Parsyan,A., Kerr,S., Owusu-Ofori,S., Elliott,G., Allain,J.P., Szmaragd,C., Allain,J.-P., Candotti,D., Parsyan,A.E.
215	AY504945.1	2004-01-21			Norja,P., Hokynar,K., Aaltonen,L.M., Chen,R., Ranki,A., Partio,E.K., Kiviluoto,O., Davidkin,I., Leivo,T., Eis-Hubinger,A.M., Schneider,B., Fischer,H.P., Tolba,R., Vapalahti,O., Vaheri,A., Soderlund-Venermo,M., Hedman,K., Kakkola,L.M., Hedman,K.P., Cohen,B.J.
216	AB126262.1	2003-11-20			Abe,K., Kiuchi,T., Tanaka,K., Edamoto,Y., Aiba,N., Sata,T.
217	AB126263.1	2003-11-20			Abe,K., Kiuchi,T., Tanaka,K., Edamoto,Y., Aiba,N., Sata,T.
218	AB126264.1	2003-11-20			Abe,K., Kiuchi,T., Tanaka,K., Edamoto,Y., Aiba,N., Sata,T.
219	AB126265.1	2003-11-20			Abe,K., Kiuchi,T., Tanaka,K., Edamoto,Y., Aiba,N., Sata,T.
220	AB126266.1	2003-11-20			Abe,K., Kiuchi,T., Tanaka,K., Edamoto,Y., Aiba,N., Sata,T.
221	AB126267.1	2003-11-20			Abe,K., Kiuchi,T., Tanaka,K., Edamoto,Y., Aiba,N., Sata,T.
222	AB126268.1	2003-11-20			Abe,K., Kiuchi,T., Tanaka,K., Edamoto,Y., Aiba,N., Sata,T.
223	AB126269.1	2003-11-20			Abe,K., Kiuchi,T., Tanaka,K., Edamoto,Y., Aiba,N., Sata,T.
224	AB126270.1	2003-11-20			Abe,K., Kiuchi,T., Tanaka,K., Edamoto,Y., Aiba,N., Sata,T.
225	AB126271.1	2003-11-20			Abe,K., Kiuchi,T., Tanaka,K., Edamoto,Y., Aiba,N., Sata,T.
226	AY386330.1	2003-10-08			Zhi,N., Zadori,Z., Brown,K.E., Tijssen,P.
227	AY083234.1	2002-08-28			Servant,A., Laperche,S., Lallemand,F., Marinho,V., De Saint Maur,G., Meritet,J.F., Garbarg-Chenon,A., Marhino,V.
228	AY064475.1	2002-05-23			Nguyen,Q.T., Wong,S., Heegaard,E.D.,

Sr. No	Accession	Release Date	Isolation Source	Collection Date	Submitters
					Brown,K.E.
229	AY064476.1	2002-05-23			Nguyen,Q.T., Wong,S., Heegaard,E.D., Brown,K.E.
230	AY044266.2	2001-09-02			Hokynar,K., Soderlund-Venermo,M., Pesonen,M., Ranki,A., Kiviluoto,O., Partio,E.K., Hedman,K.
231	AY028237.1	2001-04-14			Tolfvenstam,T., Norbeck,O., Brytting,M., Hemauer,A., Modrow,S., Broliden,K.
232	AX003421.1	2000-08-24			Auguste,V., Garbarg-Chenon,A., Nguyen,Q.T.
233	AB030673.1	2000-01-26			Umene,K., Nunoue,T., Takasawa,N., Munakata,Y., Ishii,K.K., Takahashi,Y., Takahashi,M., Fu,Y., Ishii,T., Fujii,H., Saito,T., Takano,H., Noda,T., Suzuki,M., Nose,M., Zolla-Patzner,S., Sasaki,T.
234	AB030693.1	2000-01-26			Ishii,K.K., Munakata,Y., Funato,T., Fu,Y., Koseki,N., Sugamura,K., Sasaki,T.
235	AB030694.1	2000-01-26			Takasawa,N., Munakata,Y., Ishii,K.K., Takahashi,Y., Takahashi,M., Fu,Y., Ishii,T., Fujii,H., Saito,T., Takano,H., Noda,T., Suzuki,M., Nose,M., Zolla-Patzner,S., Sasaki,T.
236	AF162273.1	1999-08-02			Gallinella,G., Venturoli,S.
237	AF161223.1	1999-07-29			Hokynar,K., Brunstein,J., Soderlund-Venermo,M., Kiviluoto,O., Partio,E.K., Konttinen,Y., Hedman,K., Brunstein,J.D.
238	AF161224.1	1999-07-29			Hokynar,K., Brunstein,J., Soderlund-Venermo,M., Kiviluoto,O., Partio,E.K., Konttinen,Y., Hedman,K., Brunstein,J.D.
239	AF161225.1	1999-07-29			Hokynar,K., Brunstein,J., Soderlund-Venermo,M., Kiviluoto,O., Partio,E.K., Konttinen,Y., Hedman,K., Brunstein,J.D.
240	AF161226.1	1999-07-29			Hokynar,K., Brunstein,J., Soderlund-Venermo,M., Kiviluoto,O., Partio,E.K., Konttinen,Y., Hedman,K., Brunstein,J.D.
241	AF113323.1	1999-01-03			Hemauer,A., Beckenlehner,K., Wolf,H., Lang,B., Modrow,S.
242	Z70528.1	1996-04-09			Hemauer,A., von Poblotzki,A., Gigler,A., Cassinotti,P., Siegl,G., Wolf,H., Modrow,S.
243	Z70560.1	1996-04-09			Hemauer,A., Von Poblotzki,A., Gigler,A., Cassinotti,P., Siegl,G., Wolf,H., Modrow,S.
244	Z70599.1	1996-04-09			Hemauer,A., Von Poblotzki,A., Gigler,A., Cassinotti,P., Siegl,G., Wolf,H., Modrow,S.
245	Z68146.1	1995-12-05			Hicks,K.E., Cubel,R.C., Cohen,B.J., Clewley,J.P.
246	M13178.1	1993-08-03			Shade,R.O., Blundell,M.C., Cotmore,S.F., Tattersall,P., Astell,C.R.
247	M24682.1	1993-08-03			Blundell,M.C., Beard,C., Astell,C.R.

Appendix 2: Details of cases positive for B19V by PCR

	ID	**BDN-18-005**	**BDN-18-046**	**BDN-18-064**	**BDN-18-195**	**BDN-18-200**	**BKR-18-160**	**BKR-18-188**	**BKR-18-202**	**BDN-17-357**	**DJL-17-157**	**BDN-17-344**	**BDN-18-017**	**BDN-18-181**
**Epidemiological data**	Sex	Male	Female	Male	Female	Male	Female	Male	Female	Male	Female	Female	Female	Male
	Age (yrs)	0.4	4	0.66	2	6	22	10	30	7	15	45	5	5
	Month of admission	January	February	March	June	June	August	September	September	November	October	November	January	May
	Date of Illness Onset	2017-12-28	2018-02-10	2018-03-14	2018-04-02	2018-06-02	2018-08-17	2018-09-02	2018-08-22	2017-11-07	2017-09-18	2017-11-05	2018-01-12	2018-05-10
	District	Darjeeling	Bardhaman	Bardhaman	Bardhaman	Bardhaman	Bankura	Purulia	Bankura	Deoghar	Darjeeling	Bardhaman	Hooghly	Bardhaman
**Clinical Presentation**	Admission diagnosis	AMES	AES	AES	Meningitis	Meningitis	AES	Meningitis	AES	AES	AES	AMES	AES	AMES
	Fever	Y	Y	Y	Y	Y	Y	Y	Y	Y	Y	Y	Y	Y
	Change in Mental status	N	Y	N	Y	N	N	Y	Y	Y	Y	Y	Y	N
	Seizure	Y	Y	Y	Y	N	Y	Y	N	Y	Y	N	Y	N
	Neck Rigidity	NA	Y	NA	Y	NA	NA	NA	NA	Y	NA	Y	Y	Y
	Drowsiness	NA	Y	NA	Y	NA	NA	NA	NA	Y	NA	NA	Y	NA
	Other symptoms	Cold, Respiratory distress		NA	history of enteric fever, lethargy	headache, backache	NA	NA	NA	Headache, vomiting	NA	Headache, cerebral edema	Headache, irritability, vomiting	rash
	CSF Glucose level (mg/dl)	66.1	125	38.3	15	60.9	ND	32	ND	ND	22	53	ND	ND
	CSF protein level (mg/dl)	73.6	50.2	51.1	258	62.4	ND	73	ND	ND	44	69	ND	ND
	CSF WBC (count/mm3)	5	5	4	14	13	ND	5	ND	ND	40	20	ND	ND
	Outcome of Hospitalization	LAMA	R&D	R&D	LAMA	R&D	R&D	R&D	R&D	R&D	Died	R&D	R&D	R&D
**Microbiological Tests***	JE IgM (CSF)	-	-	-	-	-	-	-	-	+	-	+	-	-
	JE IgM	N.D	N.D	N.D	-	-	-	N.D	-	N.D	+	+	N.D	-

	ID	**BDN-18-005**	**BDN-18-046**	**BDN-18-064**	**BDN-18-195**	**BDN-18-200**	**BKR-18-160**	**BKR-18-188**	**BKR-18-202**	**BDN-17-357**	**DJL-17-157**	**BDN-17-344**	**BDN-18-017**	**BDN-18-181**
	(Serum)													
	Scrub typhus IgM (Serum)	N.D	N.D	N.D	-	-	-	N.D	-	N.D	-	-	N.D	+
	WNV IgM (Serum)	N.D	N.D	N.D	-	-	-	N.D	N.D	N.D	-	+	N.D	-
	Leptospira sp IgM (Serum)	ND	N.D	N.D	-	-	-	N.D	N.D	N.D	-	+	N.D	-
	CHIKV IgM (Serum)	N.D	N.D	N.D	-	-	-	N.D	N.D	N.D	-	-	N.D	-
	DENV IgM (Serum)	N.D	N.D	N.D	-	-	-	N.D	-	N.D	-	+		-
	DENV NS1 (Serum)	N.D	N.D	N.D	-	-	-	N.D		N.D	-	-		-
	Malaria (Rapid test)	N.D	-	-	N.D	N.D	-	-	-	-	-	-	-	-
	SP PCR(CSF)	-	-	-	-	-	-	-	-	-	-	-	+	-
	HSV PCR (CSF)	-	N.D	N.D	N.D	N.D	N.D	N.D		-	-	-	-	N.D
	Enterovirus PCR (CSF)	-	N.D	N.D	N.D	N.D	N.D	N.D		-	-	N.D	-	N.D
	Trioplex PCR (CSF)	-	-	-	-	-	-	-	-	-	-	ND	-	-
	Microbiological/ Aetiologic alDiagnosis	AES Unknown	AES Unknown	AES Unknown	AES Unknown	AES Unknown	AES Unknown	AES Unknown	AES Unknown	JE	JE	JEV+WNV+DENV+Leptospira sp	SP	Scrub Typhus

CHIKV = Chikungunya virus, DENV = Dengue viruses, JE = Japanese Encephalitis, JEV= Japanese encephalitis virus, SP = Streptococcus pneumoniae, WNV= West Nile Virus, HSV = Herpes Simplex Virus 1, LAMA = Left against medical advice, R&D = Recovered and Discharged, AES = Acute Encephalitis Syndrome, AMES = Acute Meningoencephalitis Syndrome . *All Samples were negative for Haemophilus influenzae and Neisseria meningitidis by PCR.

Appendix 3: Description of PCR results and recovered genomes

Sample	Ct value	Filtered Reads	% Mapped	% Refseq (1X coverage)	% RefSeq (10 X coverage)	Average Depth	GenBank_Accession
BDN-17-344	27	116520	79.65	99.8	99.71	9533.45	OK482570
BDN-17-357	24	91310	79.17	99.8	99.73	7075.85	OK482571
BDN-18-005	35	27732	76.91	98.4	93.47	2864.9	OK482572
BDN-18-017	22	13559	52.28	97.4	79.9	823.73	OK482573
BDN-18-046	35	18300	58.71	99.7	98.62	1270.3	OK482574
BDN-18-064	18	123227	80.41	100	100	13540.9	OK482575
BDN-18-181	14	86150	79.95	100	100	14685.5	OK482576
BDN-18-195	18	81711	77.34	99.9	99.85	7155.4	OK482577
BDN-18-200	23	141235	79.21	100	99.87	11715.6	OK482578
BKR-18-160	22	232231	81.59	99.9	99.75	22510	OK482579
BKR-18-188	34	68234	80.87	99.8	99.69	5578.5	OK482580
BKR-18-202	30	335815	80.68	100	99.73	27908	OK482581
DJL-17-157	32	23	70.37	76.1	-	1.58	-

Appendix 4: List of unique SNPs in B19V genomes from the study

Ref Position (NC_004295)	BDN-18-017	BDN-18-005	BDN-18-046	BDN-18-344	BDN-18-064	BDN-18-181	BDN-18-200	BDN-18-195	BKR-18-188	BKR-18-202	BKR-18-160	BDN-17-357
815	N	N	**G**	C	C	C	C	C	C	C	C	C
1110	G	G	G	G	G	G	G	G	**A**	G	G	G
2142	T	T	T	**C**	T	T	T	T	T	T	T	T
2467	T	T	T	**C**	T	T	T	T	T	T	T	T
2634	G	G	**C**	**C**	G	G	G	G	G	G	G	G
2702	A	A	A	A	A	A	**G**	A	A	A	A	A
3313	N	T	**C**	T	**C**	T	T	T	T	T	T	T
3778	**C**	T	T	T	T	T	T	T	T	T	T	T
3910	G	G	G	**T**	G	G	G	G	G	G	G	G
3943	A	A	A	**T**	A	A	A	A	A	A	A	A
4255	T	T	T	**C**	T	T	T	T	T	T	T	T
4258	G	G	G	G	G	G	**A**	G	G	G	G	G
4360	**A**	G	G	**A**	G	G	G	G	G	G	G	**A**
4468	A	A	A	**G**	A	A	A	A	A	A	A	A
4531	T	T	**G**	**G**	T	T	T	T	T	T	T	T
4708	G	G	G	**A**	G	G	G	G	G	G	G	G
4758	C	C	C	C	C	**G**	**G**	C	C	C	C	C
Unique mutation count	2	0	4	10	1	1	3	0	1	0	0	1
